# The effects of disruption of phosphoglucose isomerase gene on carbon utilisation and cellulase production in *Trichoderma reesei *Rut-C30

**DOI:** 10.1186/1475-2859-10-40

**Published:** 2011-05-24

**Authors:** M Carmen Limón, Tiina Pakula, Markku Saloheimo, Merja Penttilä

**Affiliations:** 1 VTT, P.O. Box 1000, (Tietotie 2, Espoo), FIN-02044 VTT, Finland; 2 Departamento de Genética, Facultad de Biología, Universidad de Sevilla, Apartado 1095, 41080 Seville, Spain

## Abstract

**Background:**

Cellulase and hemicellulase genes in the fungus *Trichoderma reesei *are repressed by glucose and induced by lactose. Regulation of the cellulase genes is mediated by the repressor CRE1 and the activator XYR1. *T. reesei *strain Rut-C30 is a hypercellulolytic mutant, obtained from the natural strain QM6a, that has a truncated version of the catabolite repressor gene, *cre1*. It has been previously shown that bacterial mutants lacking phosphoglucose isomerase (PGI) produce more nucleotide precursors and amino acids. PGI catalyzes the second step of glycolysis, the formation of fructose-6-P from glucose-6-P.

**Results:**

We deleted the gene *pgi1*, encoding PGI, in the *T. reesei *strain Rut-C30 and we introduced the *cre1 *gene in a Δ*pgi1 *mutant. Both Δ*pgi1 *and *cre1^+^*Δ*pgi1 *mutants showed a pellet-like and growth as well as morphological alterations compared with Rut-C30. None of the mutants grew in media with fructose, galactose, xylose, glycerol or lactose but they grew in media with glucose, with fructose and glucose, with galactose and fructose or with lactose and fructose. No growth was observed in media with xylose and glucose. On glucose, Δ*pgi1 *and *cre1^+^*Δ*pgi1 *mutants showed higher cellulase activity than Rut-C30 and QM6a, respectively. But in media with lactose, none of the mutants improved the production of the reference strains. The increase in the activity did not correlate with the expression of mRNA of the xylanase regulator gene, *xyr1*. Δ*pgi1 *mutants were also affected in the extracellular β-galactosidase activity. Levels of mRNA of the glucose 6-phosphate dehydrogenase did not increase in Δ*pgi1 *during growth on glucose.

**Conclusions:**

The ability to grow in media with glucose as the sole carbon source indicated that *Trichoderma *Δ*pgi1 *mutants were able to use the pentose phosphate pathway. But, they did not increase the expression of *gpdh*. Morphological characteristics were the result of the *pgi1 *deletion. Deletion of *pgi1 *in Rut-C30 increased cellulase production, but only under repressing conditions. This increase resulted partly from the deletion itself and partly from a genetic interaction with the *cre1-1 *mutation. The lower cellulase activity of these mutants in media with lactose could be attributed to a reduced ability to hydrolyse this sugar but not to an effect on the expression of *xyr1*.

## Background

*Trichoderma reesei*, an anamorph of the species *Hypocrea jecorina*, stands out for its ability to degrade cellulose and hemicellulose. Cellulose is a long polymer of β-1,4-linked D-glucospyranose units. Degradation of cellulose to glucose requires the synergistic action of different types of enzymes. In *T. reesei*, at least several endoglucanases (EGI/Cel7B, EGII/Cel5A, EGIII/Cel12A, EGIV/Cel61A, and EGV/Cel45A), exoglucanases (the cellobiohydrolases CBHI/Cel7A and CBHII/Cel6A) and β-glucosidases (BGLI/Cel3A and BGLII/Cel1A) contribute to the total cellulase activity [1,2]. An additional protein, swollenin, disrupts crystalline cellulose structures, making polysaccharides more accessible [3].

Cellulase induction in *T. reesei *is modulated by several physiological and environmental conditions, such as growth rate [4], sulphur [5] and light [6,7]. Cellulose and disaccharides such as lactose, cellobiose and sophorose (glycosyl β-1,2-glucose) [8,9] can act as inducers and, in fact, lactose is used in industry as a cheap inducer. In *T. reesei*, lactose is hydrolysed, mainly by an extracellular β-galactosidase, Bga1, to the monosaccharides D-glucose and D-galactose which are taken up by appropriate permeases [10]. Cellulase induction by lactose requires the β-anomer of D-galactose, that cannot be catabolized via the Leloir pathway but can be converted to fructose by an alternative pathway [11]. D-xylose reductase (encoded by *xyl1*) is the enzyme that catalyzes the first step in the alternative pathway [12]. XYR1 (xylanase regulator 1) is essential for the activation of *xyl1*. This regulator is also an indispensable transcription factor for expression of hydrolytic enzymes on lactose [13,14].

The majority of the cellulase genes are repressed by the presence of glucose [15,16]. This repression is mediated by CRE1, a two-zinc finger protein that binds to DNA [17,18]. Other negatively- and positively-acting transcription factors have been characterised for cellulase and hemicellulase genes, and their functions have been described in detail [15,19-22].

Cellulases have been used for different applications in food, textile and paper industry and recently have gained interest for the production of bioethanol [19]. Demands of biofuel obtained from renewable feedstock require high amounts of cellulases [23].

One of the best cellulase producer strains is *T. reesei *Rut-C30. This strain was obtained from the natural isolate QM6a through three mutagenesis steps: (i) UV mutagenesis and screen for the ability to hydrolyse cellulose in repressing conditions; (ii) N-nitrosoguanidine mutagenesis and a similar screen; and (iii) UV mutagenesis, screen for cellulase activity and selection for 2-deoxyglucose resistance to eliminate catabolite repression [24]. The comparison between the genomes of Rut-C30 and QM6a revealed a surprisingly high number of mutagenic events [25,26]. Some of them had been previously characterized, including a truncation of the *cre1 *gene, encoding the CRE1 repressor; a frameshift mutation in the glucosidase II alpha subunit gene *gls2α*, involved in protein glycosylation [27]; and an 85-kb deletion that eliminated 29 genes, including transporters, transcription factors, and primary metabolic enzymes [28]. The truncated form of *cre1 *(allele *cre1-1*) encodes a truncated protein of only 95 amino acids with a single zinc finger [17]. This mutation, which is equivalent to a null mutation [29], produces catabolite derepression and is one of the main factors responsible for the hypercellulolytic phenotype of Rut-C30.

*T. reesei *is not only biotechnologically important for the cellulase production but also for being an efficient host for large scale production of other enzymes [30]. Different strategies have been applied to improve protein production by this fungus, including the use of strong promoters [31], induction of the unfolded-protein response [32], generation of fusions to a hydrophobic tag [33], control of the specific growth rate [4], and modification of carbon repression [29].

**Figure 1:**
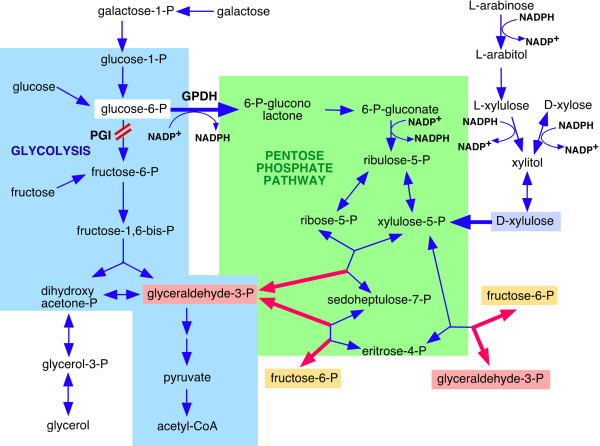
**Glycolysis and the pentose phosphate pathway**. Enzyme abbreviations: GPDH, glucose-6-P dehydrogenase and PGI, phosphoglucose isomerase.

Although Rut-C30 is a good cellulase-producer microorganism, some improvements are still possible [34]. We sought to explore possibilities to engineer the cellulase production capacity of the overproducer Rut-C30 by alteration of a metabolic pathway. The idea was to eliminate phosphoglucose isomerase (PGI) activity in order to redirect the carbon flux through the pentose phosphate pathway (PPP). The two central pathways of carbon metabolism are glycolysis and the PPP (Figure 1). The PPP is the major source of NADPH, needed for the biosynthesis of many biomolecules, in particular fats [35]. It also provides intermediates for the synthesis of amino acids: histidine is synthesised from ribose 5-phosphate (ribose-5-P), and erythrose 4-phosphate is one of the metabolic precursors needed for the synthesis of aromatic amino acids: phenylalanine, tyrosine and tryptophan [35]. For this reason, it is obvious that the PPP plays an important role in the production of proteins and therefore this pathway is expected to be relevant especially in an organism with an efficient protein production system. The PPP intermediate ribose-5-P is also essential for the synthesis of nucleotides and nucleic acids.

PGI catalyzes the second step of glycolysis converting glucose 6-phosphate (glucose-6-P) into fructose 6-phosphate (fructose-6-P). Since this enzyme is located at the first junction between glycolysis and the PPP, a consequence of the inactivation of PGI could be the rerouting of the metabolic flux through the PPP, which, in turn, would lead to an increase in NADPH production. This is, in fact, the result of the disruption of the *pgi *gene in the bacterial species *Escherichia coli *[36]. A similar result was obtained with the *pgi *mutant of the yeast *Kluyveromyces lactis *(*rag2 *mutant), which was able to grow on glucose, bypassing the glycolytic step. This suggests that glucose-6-P was metabolized via the PPP in *K. lactis *[37]. However, fungal species show differences in the capacity to use the PPP, and, in contrast with the situation in *K. lactis *and *E. coli*, the deletion of *PGI1 *in *Saccharomyces cerevisiae *prevents growth on glucose [38,39]. The *S. cerevisiae PGI1 *mutants can grow with fructose as main carbon source, but trace amounts of glucose are required [38,39] because it is the only way to provide glucose-6-P that is required for biosynthesis of many cell components. Boles *et al*. [40] proposed that the growth defect of the *PGI1 *mutants of *S. cerevisiae *on glucose medium was due to a rapid depletion of cytoplasmic NADP, which is needed as a cofactor in the PPP. This defect could be solved either by adding oxidizing agents to restore NADP from NADPH or by over-expressing the NADH-dependent glutamate dehydrogenase, resulting in substrate cycling and coupled conversion of NAD to NADH and NADPH to NADP [40]. At the same time, high concentrations of glucose prevent growth, probably due either to the toxic effect of high levels of glucose-6-P or to ATP depletion [41]. In addition, there is a great intraspecies diversity in the PPP capacity among *S. cerevisiae *strains and a low capacity may be the main factor limiting glucose oxidation through this pathway [42].

In this work, we have constructed *pgi1 *deletion mutants (Δ*pgi1*) in *T. reesei *Rut-C30 to block the early part of the glycolytic pathway and we have analysed if this mutation leads to the redirection of carbon flux through the PPP. We report the effects of this genetic alteration on morphology, growth, carbon utilisation, lactose metabolism, and, especially, cellulase production. The analysis has been carried out, both in the original *cre1-1 *background and in a *cre1^+ ^*background.

## Results

### Cloning and analysis of the *pgi1 *gene of *T. reesei*

To generate a cassette to disrupt the *pgi1 *gene of *T. reesei*, it was necessary to clone 5' and 3' fragments of the genomic sequence of the gene. Therefore, the *pgi1 *cDNA already available was used as a probe to screen a genomic library of *T. reesei *and the complete genomic sequence was isolated (see Methods). Southern hybridization under low-stringency conditions indicated that the strain Rut-C30 has a single copy of the *pgi1 *gene (data not shown). In the public genome sequence of *T. reesei *(Joint Genome Institute, USA) the *pgi1 *gene is shown as fgenesh1_pm.C_scaffold_29000020, and no other homologous ORFs are found in the *Trichoderma reesei *database v2.0 [43]. Comparison of the genomic and the cDNA sequences showed that this gene consists of 4 exons and 3 introns.

The 554 amino acids of the predicted protein product, PGI (CAG38420), were used in a BLAST search [44]. The best scores of overall amino acid identity were 87% with *Nectria haematococca *PGI (XP_003050252), 86% with PGI from *Gibberella zeae *PH-1 (XP_386019), 85% with *Verticillium albo-atrum *(XP_003009491.1), 84% with *Glomerella graminicola *(EFQ34773.1), 82% with *Magnaporthe oryzae *(XP_001408812.1), 82% with an unnamed protein of *Sordaria macrospore *(CBI53771.1), 82% with *Neurospora crassa *PGI (XP_962135), 80% with *Chaetomium globosum *(XP_001223630.1), 80% with an unnamed protein product of *Podospora anserina *(CAP65411.1), and 76% with *Aspergillus fumigatus *PGI (XP_755312.1).

### Construction of *T. reesei pgi1 *deletion strains

**Figure 2:**
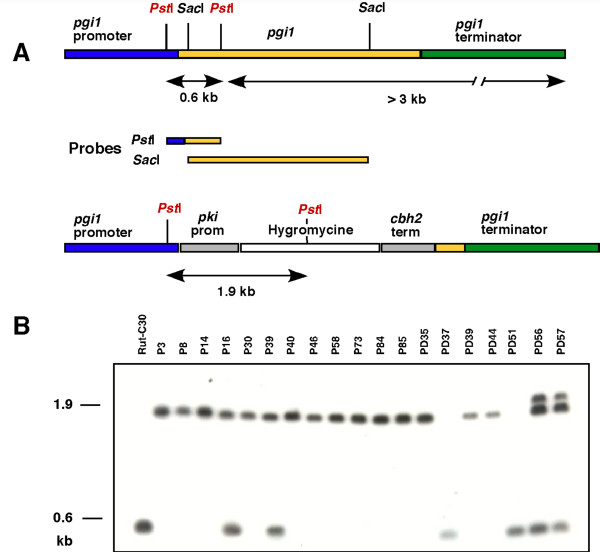
**Map and Southern analysis of Rut-C30 and putative Δ*pgi1 *mutants**. **(A) **Rut-C30 genomic *pgi1*. **(B) **Correct insertion of the deletion cassette in the Δ*pgi1 *mutants. Probes used for Southern experiments are indicated as bars that are located in the corresponding region of the DNA where they hybridize. **(C)**Southern analysis. Genomic DNA of strain RutC-30 and 19 putative transformants were digested with *Pst*I. Filter was probed with a 0.6-kb *Pst*I fragment comprising part of the coding region and part of the 5' UTR of the *pgi *gene.

A disruption cassette, containing a gene conferring resistance to hygromycin flanked by *pgi1 *promoter and terminator sequences, was generated and used to obtain the Δ*pgi1 *mutants as described in the Methods section (Figure 2A).

After transformation of *T. reesei *Rut-C30 protoplasts, 29 out of 87 resistant colonies showed poor growth and were selected as putative Δ*pgi1 *mutants. The candidates were subjected to 3 rounds of purification on minimal medium (MM) Petri dishes containing 20 g l^-1 ^fructose and 1 g l^-1 ^glucose (and Triton X-100) where they grew as small colonies. The transformants grew as well as the parental strain, however, in liquid rich PD medium with 20 g l^-1 ^fructose (data not shown). DNA isolated from the parental strain and 19 transformants were analysed by Southern blot to verify single copy insertion of the cassette at the *pgi1 *locus and correct deletion of the *pgi1 *coding region. Hybridization with a probe specific for the hygromycin resistance gene revealed only a band, indicating a single integration of the deletion cassette in the transformants (results not shown). Hybridization with a *pgi1 *specific probe (*Pst*I probe), containing part of the *pgi1 *promoter as well as the 5' end of the coding region of the gene, showed that there were different kinds of transformants (Figure 2B): (i) transformants PD37 and PD51 had the same pattern than the parental strain; (ii) transformants P16 and P39 showed two hybridizing signals, one of 0.6 kb, corresponding to the endogenous copy of the gene, and a 1.9-kb band corresponding to the expected size of the fragment in the deletion cassette; (iii) transformants PD56 and PD57 gave 3 bands; (iv) finally, 13 of the transformants gave only a signal corresponding to the disrupted gene (1.9 kb), indicating correct replacement of the gene. Lack of the original coding sequence in the last 13 candidates was confirmed by Southern blot analysis using a *pgi1 *cDNA fragment as a probe (*Sac*I probe). No band was obtained using this probe (data not shown). These candidates were considered as correct *pgi1 *deletion mutants, and further analyses were carried out with two of them, P40 and P58. The deletion was functionally confirmed because Δ*pgi1 *strains did not reveal any PGI activity in cell extracts, whereas in the parental strain the PGI activity was 3.498 ± 0.026 nkat mg^-1 ^protein. The activity was measured twice from mycelia grown in MM with glucose.

### Introduction of the *cre1 *wild-type allele (*cre1^+^*) in a *Δpgi1 *mutant

As explained in the Background section, Rut-C30, the parental strain of our Δ*pgi1 *mutants, has a deletion in the *cre1 *gene (allele *cre1-1*), which is an important factor to explain its hypercellulolytic phenotype. Presumably, this alteration in *cre1 *could have an impact in the phenotypes that we intended to study in the *pgi1 *mutants. In order to get a control for comparison and better evaluation of the effects of the *pgi1 *deletion, we generated a version of one of the mutants carrying a wild-type copy of *cre1*, allele *cre1^+^*.

**Figure 3:**
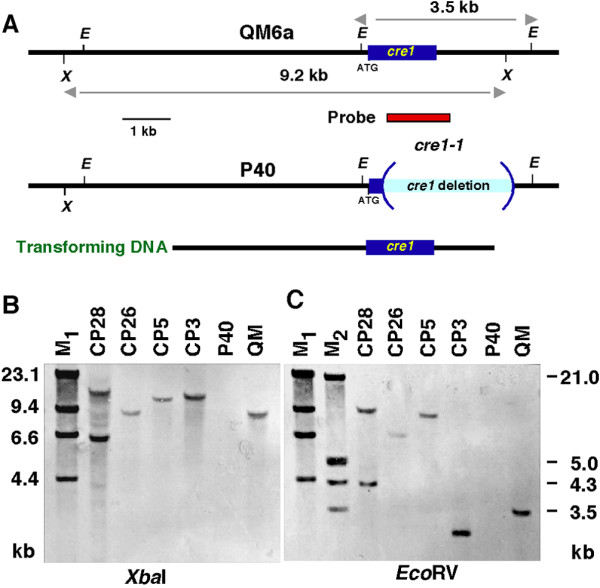
**Southern blot analysis of *cre1^+^*Δ*pgi1 *mutants**. **(A) **Map of genomic *cre1 *gene. Arrows indicate fragments released with XbaI and EcoRI that hybridize with the probe (in red). Southern analysis of strains QM6a, P40, CP3, CP5, CP26 and CP28: **(B) **Genomic DNA was digested with *Xba*I. **(C) **Genomic DNA was digested with *Eco*RV. M1 = DNA Molecular Weight Marker II, digoxigenin-labeled. M2 = DNA Molecular Weight Marker II, digoxigenin-labeled.

A 6-kb genomic fragment containing the gene *cre1 *with its own promoter was introduced in the Δ*pgi1 *mutant P40 (Figure 3A). 17 putative transformants were selected in media with acetamide, fructose and glucose. Genomic DNA was isolated from QM6a, P40 and the putative *cre1^+^*Δ*pgi1 *transformants. The DNA was digested with *Pst*I and analysed by Southern blot using a 1.2-kb DIG-labelled probe corresponding to the *cre1 *ORF. A *Pst*I 661-bp fragment of *cre1 *was not present in the allele *cre1-1*. Transformants with the inserted *cre1 *gene were identified because they showed this fragment when hybridized with a 868-bp probe specific for the region that is absent in the allele *cre1-1 *(data not shown). Four of these transformants, CP3, CP5, CP26 and CP28, were further analysed by Southern blot hybridisation. Genomic DNA of strains QM6a, P40 and the four selected *cre1^+^*Δ*pgi1 *mutants were digested with *EcoR*V, *Sac*I and *Xba*I and hybridized with the 868-bp probe. This analysis showed that CP3, CP5 and CP26 contained only one insertion of wild-type *cre1 *while CP28 contained two copies of the gene (Figure 3B). Mutants CP26 and CP28 were chosen for further analysis.

### Morphology of the Δ*pgi1 *and the *cre1^+^*Δ*pgi1 *strains

**Figure 4:**
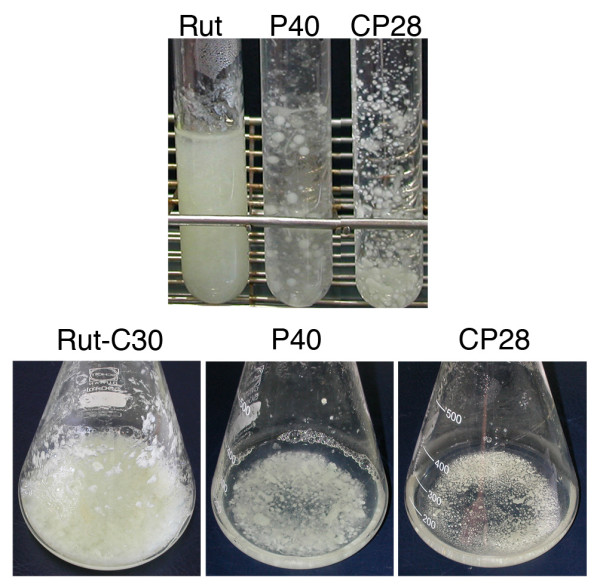
**Pellet-like growth of the mutants in liquid cultures**. Samples from the cultures and cultures of Rut-C30, P40 and CP28 that are below. Strains were grown in MM with glucose and fructose at 30°C and 150 rpm. Photos were taken after 3 days of growth.

**Figure 5:**
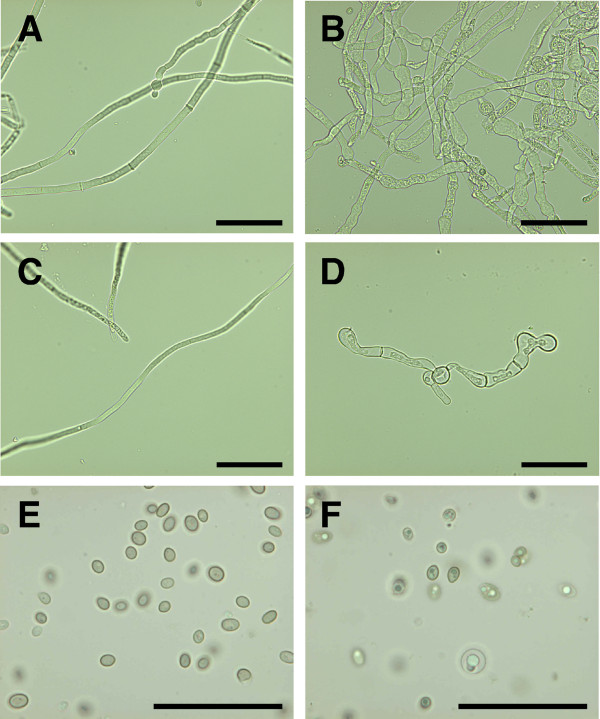
**Comparison of morphology of *T. reesei *Rut-C30 and a Δ*pgi1 *mutant**. **(A) **Rut-C30 mycelium grown in MM with glucose. **(B) **Δ*pgi1 *mycelium grown in MM and glucose. **(C) **Rut-C30 mycelium grown in MM and fructose. **(D) **Δ*pgi1 *mycelium grown in MM with fructose. Mycelia were grown for 119 h in 28°C at 200 rpm. **(E) **Conidia from Rut-C30 mycelia grown on PDA with fructose. **(F) **Conidia from a Δ*pgi1 *grown on PDA with fructose. Scale bars represent 10 μm.

First, we studied the growth and morphology of the *pgi1 *mutants in liquid and solid media. The Δ*pgi1 *and *cre1^+^*Δ*pgi1 *mutants were able to grow in liquid cultures in MM with glucose. However, differences in morphology were observed. Macroscopically, a pellet-like growth was apparent for the mutants but it was not observed for the parental strain (Figure 4). Under a microscope, the hyphae of the mutant strains were wider and more branched compared to the parental strain, and contained balloon-like structures (Figure 5A and 5B). In liquid cultures containing fructose as a sole carbon source, growth of the Δ*pgi1 *strains ceased at early stages soon after germination. On the other hand, the parental strain showed normal phenotype with long hyphae (Figure 5C) whereas the germinated hyphae of the Δ*pgi1 *and *cre1^+^*Δ*pgi1 *strains remained very short in fructose (Figure 5D and data not shown). Mutants also produced lower amounts of spores when grown on potato dextrose agar (PDA) with 20 g l^-1 ^fructose plates (data not shown). Under the microscope such spores showed dark organelles and some of them were swollen (Figure 5F) in comparison to those produced by RutC-30 (Figure 5E).

**Figure 6:**
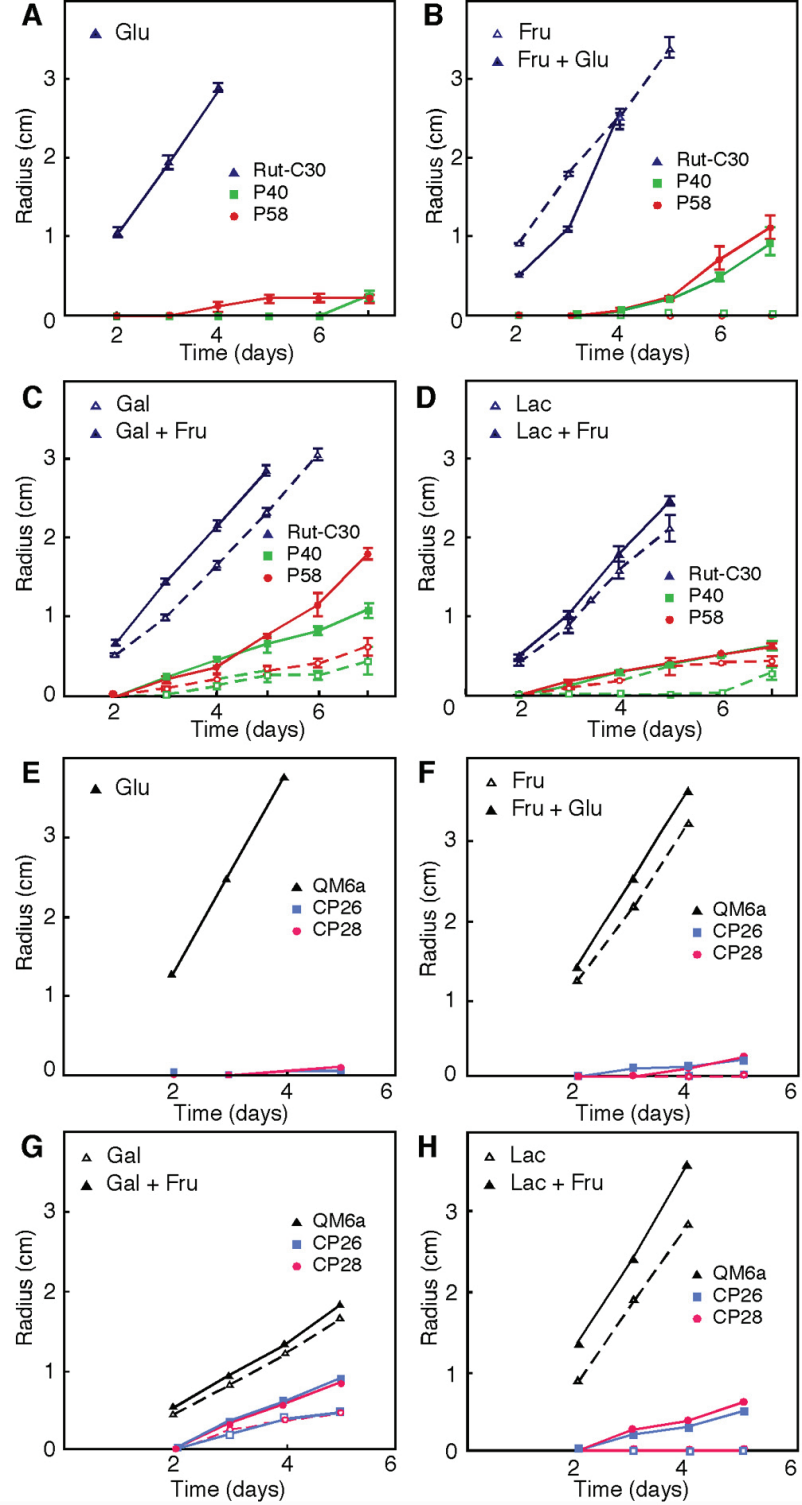
**Radial growth on different carbon sources on plates**. 7-mm plugs of Rut-C30 and Δ-*pgi1 *mutants **(A, B, C and D) **and QM6a and *cre1^+^*Δ-*pgi1 *mutants **(E**, **F, G and H) **were inoculated on Petri dishes containing: **(A and E) **MM with glucose, **(B and F) **MM with fructose and MM with fructose and glucose, **(C and G) **MM with galactose and MM with galactose and fructose, **(D and H) **MM with lactose and MM with lactose and fructose. Radial growth was measured daily during 7 days and were incubated at 22°C. The data shown are the average of three independent experiments. Error bars indicate standard deviation. (A, B, C and D) Rut-C30: blue triangles, P40: green squares and P58: red circles. QM6a: black triangles, CP26: sky blue squares and CP28: pink circles.

**Figure 7:**
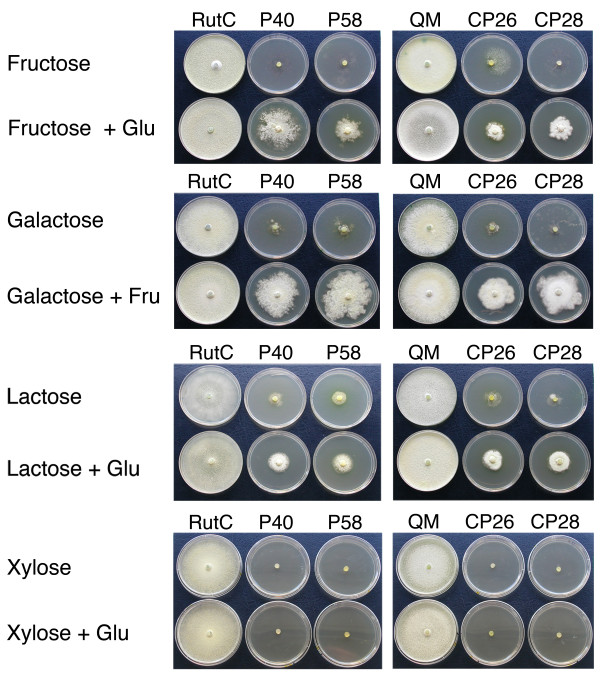
**Growth of the strains in Petri dishes with different combinations of sugars**. Panels on the left: Rut-C30, P40 and P58. Panels on the right: QM6a, CP26 and CP28. Plates were grown on minimal medium with 10 g l^-1 ^of each sugar and incubated at 22°C for 13 days.

The growth of Δ*pgi1 *and *cre1^+^*Δ*pgi1 *mutants was measured on Petri dishes with different carbon sources. On glucose as the sole carbon source, Δ*pgi1 *mutants started to grow later than in the other conditions measured (Figure 6A). Their growth on media supplemented with any of the other carbon sources along with glucose or glucose as sole carbon source was always slower than the growth of the parental strain. The Δ*pgi1 *mutants grew in media with fructose and glucose (Figure 6B), with galactose and fructose (Figure 6C), and with lactose and fructose (Figure 6D). When grown on plates of medium with fructose and glucose, the Δ*pgi1 *mutants did not show aerial hyphae and, compared to the parental strain, the mutant colony morphology was thinner at the colony edges. The diameters of the Δ*pgi1 colonies *were larger (Figure 6C) and their mycelia were thicker (Figure 7) on plates with galactose and fructose than on the other sugar combinations tested. Colony shape of Δ*pgi1 *on lactose and fructose was more symmetrical than in any of the other combinations tested. Δ*pgi1 *mutants also grew in media with glycerol and glucose although mycelia of all the strains tested, including Rut-C30, were less compact (data not shown). No growth was observed on MM plates with xylose and glucose (Figure 7). When galactose or lactose was used as the sole carbon source only a weak growth was observed after 13 days (Figure 7). The radial growth of the *cre1^+^*Δ*pgi1 *mutants was comparable to the radial growth of the Δ*pgi1 *mutants in most of the sugar combinations (Figure 6 E, F, G, H and Figure 7) and they did not grow on either xylose or xylose and glucose (Figure 7).

The conclusion of this section is that carbon sources that feed to the metabolism after the PGI step in glycolysis, i.e., fructose or glycerol, did not support growth of the mutants, but growth was restored if glucose was added to the media (Figure 6). Moreover, Δ*pgi1 *did not grow on other sugars such as, lactose or galactose, unless fructose was also added. In summary, for the conditions tested, glucose was essential for the growth of the mutants but was not enough for normal growth. In addition, the effects of the *pgi1 *mutation on growth and morphology were independent of the *cre1-1 *mutation.

### Cellulase activity

**Figure 8:**
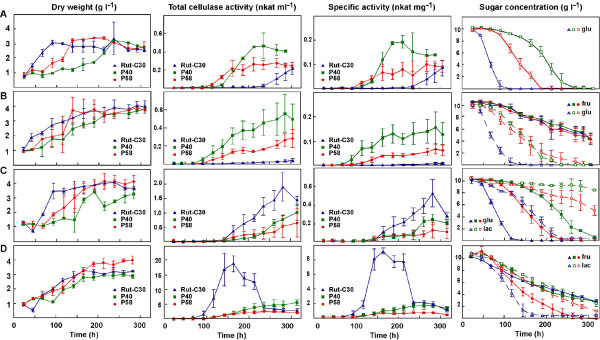
**Growth, sugar concentration and cellulase activity of Rut-C30 and Δ*pgi1 *mutants**. **(A) **MM with glucose, repressing conditions. **(B) **MM with glucose and fructose. **(C) **MM with lactose and glucose. **(D) **MM with lactose and fructose. Rut-C30: blue triangles, P40: green squares and P58: red circles. Code for sugar concentration is indicated in each graphic. 1 nkat = 1 mmol of methylumbelliferyl released from MUL per second. Cultures were grown at 30°C and 200 rpm. Triplicates of each strain were cultured for each sugar condition. Error bars indicate standard deviation.

Next, we investigated the impact of the *pgi1 *mutations on cellulase production of *T. reesei*. The Δ*pgi1 *mutants produced higher total cellulase activity than Rut-C30 in MM with glucose (Figure 8A). The maximum levels for P40 and P58 were 0.46 nkat ml^-1 ^and 0.27 nkat ml^-1^, respectively, and the maximum level for the parental strain was 0.21 nkat ml^-1^. The beginning of cellulase production in the cultures of the mutants coincided with the beginning of glucose consumption and the production ceased when the biomass reached the maximum level in the cultures. However, in Rut-C30 cellulase activity started to be detected much later, after 235 h of culture.

The mutants also produced higher cellulase activity than Rut-C30 in MM with glucose and fructose (0.56 nkat ml^-1 ^and 0.28 nkat ml^-1 ^in the *pgi1 *mutants *vs *0.04 nkat ml^-1 ^in Rut-C30) and the activity level did not decrease during the cultivation (Figure 8B). Cellulase production in the mutants started slightly earlier in MM with fructose and glucose than in MM with only glucose but maximal production was similar in both media. In contrast, Rut-C30 had almost undetectable activity in this medium. In conclusion, the cellulase production by the Δ*pgi1 *mutants exceeded the derepressed level of cellulase production in the parental strain.

Lactose is commonly used as an inducing carbon source for cellulase production and, consistent with that, higher cellulase activity levels were obtained in the presence of lactose. Rut-C30 produced 2-fold more activity than P40 mutant and 3-fold more than P58 mutant in this medium (Figure 8C). Rut-C30 reached 1.9 nkat ml^-1 ^of total cellulase activity, whereas maximal cellulase activities for P40 and P58 were 1 nakt ml^-1 ^and 0.6 nkat ml^-1^, respectively. Cellulase activity started to be detected in the three strains after 6 days of growth and increased with time.

All the strains tested exhibited increased cellulase activity in MM with lactose and fructose, in comparison to medium with lactose and glucose. However, whereas in Rut-C30 a 10-fold increase was observed, in the Δ*pgi1 *strains the activity increased about 4 to 5-fold (Figure 8D). Cellulase activity of Rut-C30 in MM with lactose and fructose was detected earlier than in MM with lactose and glucose. After reaching a maximum of 18 nkat ml^-1 ^at 142 h, there was a steep decline to 2.5 nkat ml^-1 ^at 236 h. This correlated with a decrease in extracellular protein concentration (Additional file 1B). Production was kept up until the end of the cultivation. However, in MM with lactose and glucose the decrease in activity occurred much later, after 280 h (Figure 8C). Δ*pgi1 *mutants produced a maximum of 2-5 nkat ml^-1 ^after 315 h of incubation in MM with lactose and fructose and the activity did not decrease during the cultivation. Interestingly, mutant P40 displayed more cellulase activity than Rut-C30 in the last days of incubation in this medium.

Cellobiohydrolase and endoglucase activities contributed equally to total cellulase activity measured in Δ*pgi1 *mutant filtrates from MM with glucose and MM with fructose and glucose (data not shown).

Specific cellulase activity of the Δ*pgi1 *mutants showed a similar trend to total cellulase activity in all the conditions tested, except in MM with lactose and fructose. In this medium, although P40 had higher total cellulase activity than Rut-C30 in the later stages of the cultures, between 250-300 h, (Figure 8D), the specific activities were similar for both strains. This result could be a consequence of the decrease in biomass observed for Rut-C30 at the end of the culture.

In order to evaluate the impact of CRE1 functionality on the increase in cellulase activity observed for the Δ*pgi1 *mutants, the *cre1^+^*Δ*pgi1 *mutants were analysed. Cellulase activity of *cre1^+^*Δ*pgi1 *mutants was measured both in MM with glucose and fructose and in MM with lactose and fructose, after 7 days of culture. (Table 1). Cellulase activities of the *cre1^+^*Δ*pgi1 *transformants, CP26 and CP28, in MM with glucose and fructose were lower than the cellulase activities of P40 and Rut-C30 (Table 1). This result was expected and it indicated that the introduced *cre1^+ ^allele *was functional and that its product, CRE1, was repressing cellulase genes. The comparison of *cre1^+^*Δ*pgi1 *mutants to QM6a showed that cellulase activity was higher in *cre1^+^*Δ*pgi1 *than in QM6a, indicating that the *pgi1 *mutation produced, *per se*, a hypercellulolytic phenotype. In addition, the effect of this mutation was exacerbated in a *cre1-1 *background (compare the values for P40 and CP26 in Table 1). However, in MM with lactose and fructose the cellulase activities of Δ*pgi1 *and *cre1^+^*Δ*pgi1 *mutants, were lower than the respective activities of RutC-30 and QM-6a. In conclusion, deletion of gene *pgi1*, both in a *cre1-1 *and a *cre1*^+ ^background, caused an increase in cellulase activity in medium with glucose and a drop of the activity in medium with lactose.

**Table 1:** Cellulase activity (nkat ml^-^^1^) under repressing and inducing conditions

Strains	Glu and Fru	Lac and Fru
QM-6a	0 ± 0.01	5.21 ± 0.43
RutC-30	0.61 ± 0.05	5.95 ± 0.68
P40	1.44 ± 0.60	3.48 ± 0.66
CP26	0.29 ± 0.17	1.22 ± 0.41
CP28	0.33 ± 0.17	2.66 ± 0.26

Activity was measured from samples taken after 7 days of incubation in MM with glucose and fructose and MM with lactose and fructose. Errors indicate standard deviation (n = 6).

### Growth, sugar utilisation and β-galactosidase activity of the Δ*pgi1 *mutants in liquid cultures

In order to understand the changes in cellulase activities caused by the *pgi1 *mutation in media with different carbon sources, we next analysed the utilisation of sugars by the strains under study.

As expected, glucose was uptaken more slowly by Δ*pgi1 *mutants than by Rut-C30, P40 being the slowest one. As a consequence, the mutants had longer lag and log phases of growth compared to the parental strain Rut-C30 (Figure 8A).

In cultures containing glucose and fructose, the growth of the mutants and the parental strain Rut-C30 were similar, but there were clear differences in the sugar utilisation (Figure 8B). Rut-C30 consumed glucose faster than the *pgi1 *mutants. Consistently, glucose was depleted after 100 h of cultivation of Rut-C30, whereas there were still 8.3 and 5.3 g l^-1 ^left of the sugar in the culture of P40 and P58, respectively, at the same time (Figure 8B). In this media, differences between both mutants in glucose consumption were reduced in comparison to media with only glucose. Fructose consumption was similar in all the strains.

In MM with lactose and glucose, the latter was consumed before lactose by all the strains (Figure 8C). Glucose was consumed at slightly lower rates by mutants P40 and P58, and the consumption was initiated 4 and 2 days later than by the parental strain, respectively. Rut-C30 consumed glucose completely in 120 h, and after depletion of glucose, lactose consumption increased (Figure 8C). Moreover, P58 almost did not consume lactose. Mutant P40 consumed only a small percentage of the lactose present in the culture, and this mutant consumed the lowest amount of both sugars.

In MM supplemented with lactose and fructose, lactose disappeared faster from the culture of the parental strain Rut-C30 than from the cultures of the Δ*pgi1 *mutants, whereas fructose was consumed at higher specific rate by the *pgi1 *mutants compared to the parental strain (Figure 8D). As a result, lactose remained longer in the cultures of the mutants than in the culture of Rut-C30.

When comparing sugar preferences, Rut-C30 preferred glucose to lactose in MM with lactose and glucose (Figure 8C), but in MM with lactose and fructose, Rut-C30 preferred lactose to fructose (Figure 8D). In the latter media, both sugars were detected at the same concentration in the P40 culture.

Δ*pgi1 *mutants in MM with lactose and glucose consumed lactose very slowly and most of this sugar was not used, while RutC-30 consumed completely both sugars in 250 h. At the end of the experiment, all the mutants reached similar biomass, which could seem contradictory (similar biomass was produced with less sugar) (Figure 8C). However, when we compared the extracellular protein produced in this medium (Additional file 1, Figure S1A) we noticed that RutC-30 produced over twofold the amount of protein secreted by the mutants at 220 h. The lower capacity of Δ*pgi1 *mutants to produce extracellular protein could be explained by the lower amount of sugar that entered the cells (Figure 8C).

We noticed that P40 almost did not consume lactose, produced less biomass and started to produce extracellular protein and cellulases later than P58. However, P58 consumed half of the total amount of lactose in the culture medium and produced more biomass than P40, probably as a consequence of the additional energy supply. In medium with lactose and fructose, P58 consumed fructose quicker than P40. On the other hand, P58 got higher mycelial dry weight than P40 and than the parental strain.

Because consumption of lactose in *Trichoderma *needs the extracellular hydrolysis of this sugar to galactose and glucose, we measured galactose concentration and β-galactosidase activities in supernatant samples from cultures with lactose. In most of the samples, galactose was not detected and in a few of them it was detected at very low level (maximal galactose concentration detected in Rut-C30 supernatants from MM with lactose and glucose was 0.05 g l^-1 ^at 68 h). β-galactosidase activities, measured after 168 h of growth in media with fructose and lactose, were 9.76 mU ml^-1 ^for Rut-C30, 1.98 mU ml^-1 ^for P40 and 0.88 mU ml^-1 ^for P58. The low β-galactosidase activites of the *pgi1 *mutants explain their low capacity of utilisation of lactose and the absence of galactose in the culture supernatants.

### Expression of *gpdh*, *xyr1 *and *xyl1 *in *Δpgi1 *mutants

**Figure 9:**
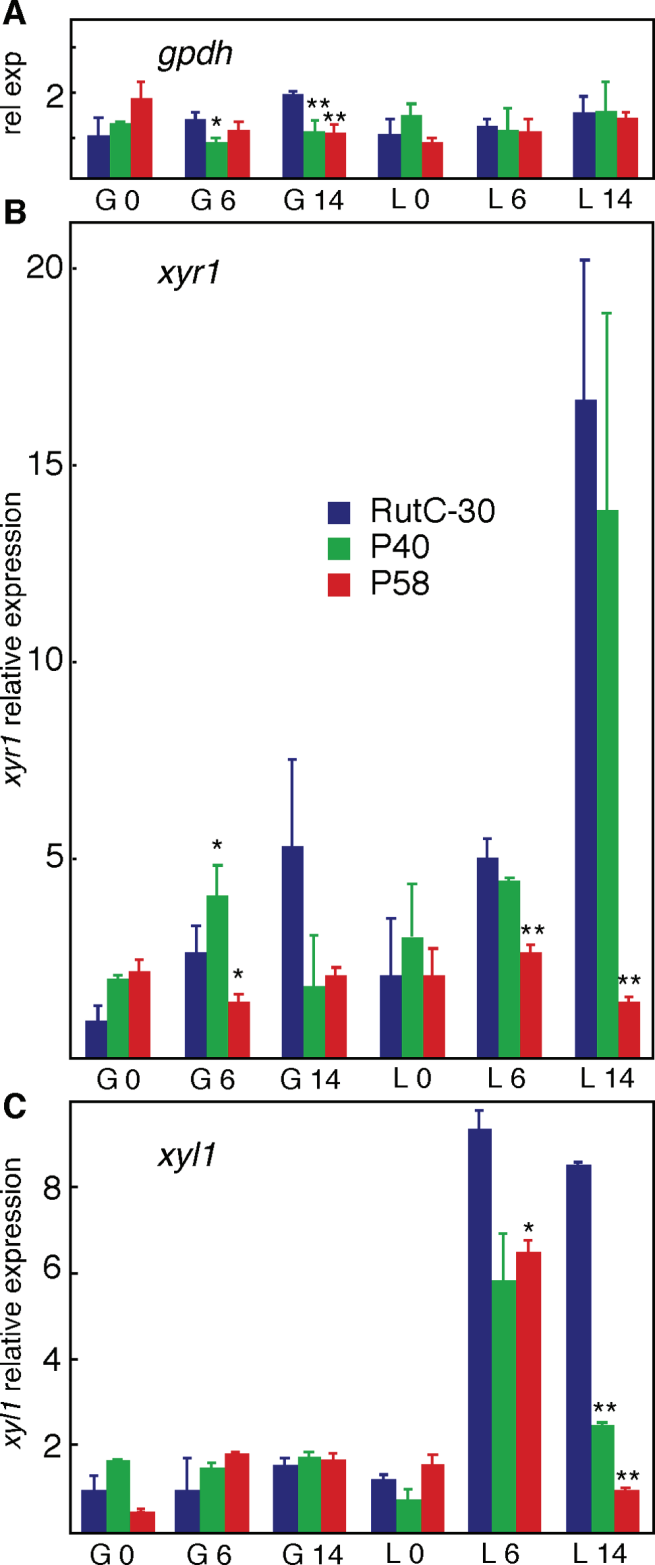
**Gene expression analysis**. **(A) **Relative expression of *gpdh*. **(B) **Relative expression of *xyr1*. **(C) **Relative expression of *xyl1*. Strains were first grown in 500-ml flasks containing 100 ml of MM with 10 g l^-1 ^glucose and 10 g l^-1 ^fructose with 2 g l^-1 ^of peptone for 30 h at 150 rpm and 30°C. Mycelia were transferred to MM containing either glucose or lactose. Samples taken after 0, 6 and 14 h of culture from glucose media are indicated as G0, G6 and G14. Samples from lactose at the same times are named L0, L6 and L14. Two independent experiments were done. Error bars indicate standard deviation. Each strain is represented by a different colour. Rut-C30: blue, P40: green and P58: red. Bars with 1 asterisk (*) are significantly different to Rut-C30 (*P *< 0.05) and bars with 2 asterisks (**) are extremely significantly different to Rut-C30 (*P *< 0.001).

Δ*pgi1 *mutants were expected to redirect the carbon flux through the PPP. Therefore, we measured the relative amount of mRNA for glucose 6-phosphate dehydrogenase (GPDH), the enzyme that converts glucose-6-P into 6-P-gluconolactone and connects glycolysis to the PPP (Figure 1). Levels of *gpdh *mRNA in the Δ*pgi1 *mutants were not higher than in Rut-C30, but they were even significantly lower in some conditions (Figure 9A). Differences between Rut-C30 and P40 were significant after 6 h of transfer to a media with glucose (*P *< 0.05). Differences between Rut-C30 and Δ*pgi1 *were statistically significant at 14 h after the transfer (*P *< 0.001). These low *gpdh *mRNA levels could lead to an accumulation of glucose-6-P that would explain the poor growth of the mutants.

As shown above, Δ*pgi1 *mutants produced more cellulase activity than the parental strain in repressing conditions but less in inducing conditions. To understand this result, we measured the expression of *xyr1*, the gene encoding the transcriptional regulator of cellulase genes XYR1, and of *xyl1*, encoding the aldose reductase XYL1. As shown in Figure 9B and 9C, mRNA levels for both genes in the *pgi1 *mutants were either similar to or lower than the levels in the parental strain. A statistically significant increase was only observed for P40 mutant after 6 h in glucose (Figure 9B). Some interesting conclusions arise from these data: (i) the low expression of these genes on lactose, relative to the parental strain, correlated with a lower cellulase production (Figure 8C and 8D); (ii) the increased cellulase production of the mutants on glucose is not totally explained by the expression levels of *xyr1 *and *xyl1*; (iii) P40 and P58 mutants showed significant differences in *xyr1 *expression (see Discussion).

## Discussion

### Construction, viability and morphology of *T. reesei pgi1 *mutants

In spite of the difficulties previously reported for *Aspergillus niger *[45], we have been able to delete the gene *pgi1 *in *T. reesei *RutC-30 by homologous recombination. We obtained single integration in 15% of the transformants and two of them, P40 and P58, have been characterized.

The *T. reesei *Δ*pgi1 *and *cre1^+^*Δ*pgi1 *mutants, although viable, showed reduced growth and produced less biomass, especially when glucose was the only carbon source. This is not surprising, because the phenomenon of the reduced growth has been observed in *pgi *mutants of many other organisms including *E. coli *[36], *Corynebacterium glutamicum *[46] and *S. cerevisiae *[40]. A striking phenotype of the Δ*pgi1 *mutants was the pellet-like growth that was observed in MM with glucose and in MM with glucose and fructose (Figure 4).

As observed under a microscope, the mycelial morphology of Δ*pgi1 *mutants in media with glucose is reminiscent of the morphology of mutants affected in the production of cell wall. In fact, Upadhyay and Shaw [47] described the morphological effects of a mutation in *swoM1*, the gene encoding PGI in *Aspergillus nidulans*. *swoM1 *mutants were not able to maintain cellular polarity and produced swollen cell apices that often lysed [47]. The authors attributed these phenotypes to the role of PGI in cell wall construction as some products downstream of PGI provide intermediates for its synthesis. In addition, the balloon-like structures and swollen hyphae that we observed in our Δ*pgi1 *mutants (Figure 5B) were also described in a chitin synthase deficient mutant of *Fusarium oxysporum *[48,49], and in a *gfdA *(encoding glycerol 3-phosphate dehydrogenase) null mutant of *A. nidulans *[50]. Phosphomannose isomerase (PMI) mutants also showed abnormal morphology with "ballooned" hyphal tips and basal cells and exhibited cell cycle alterations [51].

One possible mechanism connecting glycolysis to growth rate could be the availability of building block precursors produced by glycolysis and the PPP. Chitin, one of the main components of the fungal cell wall, is made of monomers of N-acetyl-glucosamine (GlcNAc) which is the precursor of UDP-GlcNAc. Considering that this sugar nucleotide is synthesized from fructose-1,6-P [52], low fructose-6-P concentration would affect cell wall biogenesis. Fructose-6-P can be obtained from glucose through the PPP. However, if glucose uptake is low, glucose-6-P has to provide α-glucan for cell wall synthesis and at the same time, glucose-6-P has to enter the PPP to provide NADPH for lipid synthesis, ribose for synthesis of nucleic acid, fructose-6-P for chitin and mannose-6-P for mannose-containing sugars chains. Low PGI activity has been reported in *S. cerevisiae pmi *mutants, which lack the enzyme that converts fructose-6-P to mannose-6-P. These mutants were viable only if fed with mannose and glucose. A result of the increase of mannose concentration in the cultures of these mutants was the accumulation of mannose-6-P that provoked a decrease in the flux through glycolysis [53]. In these conditions, *pmi *mutants down-regulated several genes encoding proteins involved in cell wall organization and biogenesis [53].

Our *pgi1 *mutants did not grow in MM with fructose as the sole carbon source but were able to grow if glucose was added to the culture medium (Figure 6B, F and 7). A similar result was observed when using glycerol instead of glucose. Also, *pgi *mutants of *S. cerevisiae *only grew when low amounts of glucose were supplied together with fructose (Figure 6D, H and 7). In *S. cerevisiae*, PGI is the only enzyme that could catalyse the conversion of fructose-6-P into glucose-6-P because the PPP is not active. The precursor of UDP-glucose, a substrate needed for glucan synthesis and essential for cell wall biogenesis, is glucose-6-P [51]. In addition, NADPH generated when glucose-6-P is metabolised via the PPP is required for biosynthesis of phospholipids that are essential for membranes [38].

Δ*pgi1 *and *cre1^+^*Δ*pgi1 *mutants showed the largest diameter growing on MM with galactose and fructose (Figure 6C, 7). This could be explained by the assimilation of galactose by an alternative pathway [11]. However, the Δ*pgi1 *mutants displayed shorter diameters on plates with MM with lactose and fructose than in MM with galactose and fructose (Figure 6B, F and 7). The low levels of extracellular β-galactosidase activities found in the mutants could explain this result, since this activity is necessary to convert lactose into glucose and galactose, the first step for the utilisation of lactose as a carbon source.

Mutants did not grow in media with xylose and glucose probably because xylose needs NADPH to be converted to xylitol (Figure 1). Even if GPDH could produce NADPH during the conversion of glucose to 6-P-gluconolactone (Figure 1), this NADPH probably was consumed by xylose before entering the PPP, and was not enought to maintain growth.

The phenotypes associated to the *pgi1 *mutation that are discussed in this section (viability, growth with different carbon sources, pellet-like growth, mycelial morphology, radial growth) were studied both in the parental *cre1-1 *background and in an isogenic *cre1^+^*background (Figures 4, 5, 6 and 7). The results were similar in both cases. Therefore, these phenotypes are the result of the *pgi1 *deletion and are not influenced by *cre1*.

### Effect of the deletion on total cellulase activity

Cellulase production in *T. reesei *is repressed by glucose. Cellulase activity of the strain RutC-30 in glucose-containing media is partially due to a mutation in the glucose repressor gene *cre1 *[17]. Interestingly, the Δ*pgi1 *mutation significantly increased the derepressed level of cellulase production of the parental strain RutC-30 (in MM with glucose or MM with fructose and glucose). The increase in activity is partially explained by the *pgi1 *deletion *per se*, since some increment was also observed in a *cre1^+^*background. However, the increase was relatively higher in the *cre1-1 *background. This result suggests that the effects of both mutations are not simply additive but that some kind of synergistic interaction exists between them.

The lower growth rate produced by the *pgi1 *deletion, both in *cre1-1 *and *cre1^+^*backgrounds, could explain, at least in part, the increment in cellulase activity that is specifically due to this mutation. It has been previously shown that the production of extracellular proteins in *T. reesei *is favoured at low specific growth rates [4]. The highest specific rates of cellobiohydrolase I synthesis and secretion were obtained at a specific growth rate of 0.031 h^-1^, and at higher specific growth rates both the specific synthesis and the specific secretion rates decreased [4].

Cellulase activity levels of the Δ*pgi1 *mutants in MM with lactose and fructose (Figure 8D) were higher than in MM with lactose and glucose (Figure 8C). Similarly, maximal cellulase activity of Rut-C30 was higher in MM with lactose and fructose than in MM with lactose and glucose. Both results suggest that, in spite of the *cre1-1 *mutation, glucose provokes some repression of cellulase activity when added along with lactose in comparison to the addition of fructose.

The decrease in cellulase activity exhibited by Rut-C30 in MM with lactose and fructose (Figure 8D) correlated with a progressive reduction in extracellular protein concentration after 142.8 h (Additional file 1B). Extracellular proteases have been reported to increase in response to carbon starvation in *A. nidulans *strains lacking a functional copy of *creA *[54]. Moreover, a decrease in the amount of total extracellular protein coincided with the disappearance of lactose in the culture medium, which may indicate that carbon starvation could be the explanation in the case of Rut-C30 (Additional file 1B).

The improvement produced by the *pgi1 *deletion in the level of cellulase activity under repressing conditions was not observed under inducing conditions (in lactose-containing media). In fact, the mutants exhibited 2 to 3-fold less activity in media with lactose and glucose and 3 to 9-fold less in media with lactose and fructose, when compared with the parental strain RutC-30. The low β-galactosidase activities of the mutants could explain the reduced effect of lactose, since, as mentioned in the Background section, cellulase induction by lactose requires the β-anomer of D-galactose, that is produced after the hydrolysis of lactose to D-glucose and D-galactose. In addition, the lower production of extracellular proteins in the *pgi1 *mutants in MM with lactose and fructose (Additional file, Figure S1B) could also be explained by the slow hydrolysis of lactose (Figure 8C), due to a low β-galactosidase activity.

### Sugar utilisation

Glucose consumption rate of the Δ*pgi1 *strains was reduced, in comparison with the parental strain Rut-C30, when grown in MM with glucose. In these mutants, glucose should be metabolized by the PPP instead of by glycolysis. However, glucose-6-P could be accumulated in the cells for two reasons: (i) the PGI step is blocked and glucose-6-P cannot be converted to fructose-6-P and (ii) the flux through the PPP is too slow. *E. coli *produces a glucose transporter that is downregulated upon accumulation of a high concentration of hexose-phosphate [55]. This downregulation modulates glucose uptake to avoid over accumulation of glucose-6-P and fructose-6-P, which are potentially toxic to the cell [56,57]. One possible explanation for the reduction of glucose consumption rate in the *Trichoderma *Δ*pgi1 *mutants could be a similar feedback regulation on glucose transporters. If glucose-6-P were metabolized slowly through the PPP in the mutants, glucose-6-P would be accumulated and would inhibit expression of glucose transporters.

When mutants were grown with a combination of sugars: MM with fructose and glucose or MM with fructose and lactose, their fructose consumption was faster than that of Rut-C30. Fructose must be rapidly transported and metabolized through glycolysis.

As previously discussed, the slow consumption of lactose by the *pgi1 *mutants and the differences detected in lactose concentration in the cultures of the different strains, could be explained by differences in β-galactosidase activities.

### Gene expression

*gpdh *mRNA levels did no increase in the mutants growing on glucose (Figure 9A), a result that correlated with the GPDH activity measurements (data not shown). This result suggests that glucose was not inducing *gpdh*. However, it cannot be discarded that the PPP fluxes were not regulated at *gpdh *level.

The relative expression of the cellulase activator gene, *xyr1*, is higher in the Δ*pgi1 *mutants after the transfer to glucose-containing media, which could help to explain the differences in cellulase activity under conditions of catabolite repression. The almost undetected activity in Rut-C30 cannot be explained by the mRNA levels of the activator. However, after several hours of incubation in media with glucose, Rut-C30 and the Δ*pgi1 *mutants did not show significant differences of *xyr1 *expression, except for a transient increase in P40 after 6 h of incubation in glucose (Figure 9B). Therefore, as already mentioned in the Results section, the differences observed in cellulase activities between Rut-C30 and P40 could not be totally explained by the expression of the regulator *xyr1*.

In lactose, *xyr1 *relative expression was not statistically different between P40 and Rut-C30, but the expression in P58 was significantly lower (*P *< 0.001). In Rut-C30 and P40 the *xyr1 *expression increased with time on lactose, as described for a *Δcre1 *mutant and for Rut-C30 [58]. The amount of *xyr1 *mRNA in the strain P58 started to decrease after 6 to 14 hours of incubation in media with lactose.

There were no significant differences in the expression of *xyl1 *when the different strains were grown in media with glucose (Figure 9C). However, on lactose the *xyl1 *expression was lower in the Δ*pgi1 *mutants than in Rut-C30. The differences were statistically significant for P40 and P58 at 14 h after the transfer (*P *< 0.001). This result was unexpected because XYR1 regulates the expression of *xyl1 *on lactose [14]. In this case, there should be other factors affecting the expression of *xyl1*.

The differences in β-galactosidase activity found between Rut-C30 and the Δ*pgi1 *mutants agree with the accumulation of *xyl1 *mRNA in the strains that have been studied. The enzyme D-xylose reductase, XYL1, converts D-galactose into galactitol [12]. It was formerly shown that galactitol induces β-galactosidase [14,59]. Our results are in agreement with previous data, because the mutant P58, that exhibited a reduced expression of *xyl1*, had lower β-galactosidase activity.

### Differences between *Δpgi1 *mutants P40 and P58

The Δ*pgi1 *strains analysed in this work, P40 and P58, exhibited some differences in the parameters that were measured. For instance, they differed in cellulase production, consumption of glucose and other sugars, patterns of growth, and expression of *xyr1 *and *xyl1 *under certain conditions. Although the deletion of *pgi1 *was the same in both mutants, according to Southern analysis, the phenotypic differences are indicative of some additional genotypic alteration. This is not surprising if we take into account the poor growth of the mutants in the selective medium. A consequence could be the accumulation of spontaneous suppressor mutations that could partially alleviate the growth defect. This was the case of *pgi1 *suppressor mutants isolated in *S. cerevisiae *that enhanced glucose catabolism through the PPP [40,60,61]. A suppressor gene was *GDH2 *coding for the NAD-dependent glutamate dehydrogenase [40]. The possibility of suppressor mutations made advisable the analysis of more than one independent mutant to get general conclusions about the effects of the *pgi1 *deletion. In fact, the behaviour of both mutants was similar, although P40 showed more differences than P58, when compared with the parental strain, for certain parameters. Interestingly, the main conclusions of this work (i.e., growth phenotypes, cellulase production, sugar consumption) were confirmed in both mutants.

## Conclusions

We have deleted the *pgi1 *gene in the hypercellulolytic *T. reesei *mutant Rut-C30. The resulting strains were able to grow using glucose as the only carbon source, suggesting that the PPP is active in *T. reseei*. These Δ*pgi1 *mutants exhibited higher total cellulase activity under repressing conditions in comparison to Rut-C30. The increase in activity is partially explained by the *pgi1 *mutation, and partially by to the genetic interaction between the *pgi1 *mutation and the *cre1-1 *mutation that was already present in RutC-30. In contrast, the morphological and growth alterations observed in the mutants may be attributed to the *pgi1 *mutation since similar effects were observed in *cre1^+^*and *cre1-1 *backgrounds. In summary, since the *pgi1 *mutation produces an increase in cellulase activity in certain media, even though not under inducing conditions, it could be interesting to use this mutation, in combination with others, to improve the hypercellulolytic phenotypes of *T. reesei *strains. Overexpression of *bga1 *gene could be explored as a way to increase induction by lactose in *pgi1 *mutants. Another strategy of improvement could be the co-cultivation of Δ*pgi1 *mutants with overproducer strains under inducing conditions, taking advantage of their different sugar preferences and their different production profile.

## Methods

### Strains

*T. reesei *strain Rut-C30 (ATCC 56765, VTTD-86271) [62] was used for gene disruption, strain VTT-D-80133 [63] was used for the preparation of a genomic cosmid library and the natural isolate QM6a (ATCC13631, VTT-D-071262T) was used as a control. *Escherichia coli *strains JS4 and DH5α were used for library and plasmid constructions, respectively, while strain TOP10F' was used as a host for cloning PCR products in the TOPO vector.

### Media and culture conditions

Conidia of *T. reesei *Rut-C30, *pgi1 *and *cre1^+^*Δ*pgi1 *mutants for inoculating liquid cultures were collected from mycelia grown for 7 days at 28°C on potato dextrose agar (PDA) and PDA supplemented with 20 g l^-1 ^fructose, respectively. Unless otherwise stated, the liquid cultures were grown on minimal medium (MM) [8] with 10 g l^-1 ^of each carbon source added (either glucose, fructose, lactose or a combination of them). Conidia (4 × 10^7^) of Rut-C30 or Δ*pgi1 *strains were inoculated in 400 ml of medium in 2-liter flasks and incubated on a rotary shaker at 200 rpm at 28°C. For determination of dry weight, two sample aliquots were weighed and collected on pre-weighed filter disks (Whatman GF/B 55 mm Ø). The biomass was washed with approximately 50 ml of demineralised water and dried overnight in an oven at 105°C, and afterwards the disks were weighed again. Triplicates of each strain were cultured for each condition.

For comparison of cellulase activity among all the strains, Rut-C30, QM6a, Δ*pgi1 *and *cre1^+^*Δ*pgi1 *mutants were cultured in 500-ml flasks containing 100 ml of MM with glucose and fructose or MM with lactose and fructose. Mycelia were incubated for 7 days at 30°C in a rotary shaker at 150 rpm. β-galactosidase activity was measured from these culture supernatants.

For gene expression experiments, strains were first grown in 500-ml flasks containing 100 ml of MM with glucose, fructose and 2 g l^-1 ^peptone at 150 rpm and 30°C. After 30 h of culture, mycelia from each strain were filtered, washed and equal amounts of each strain were resuspended in MM containing glucose or lactose. Samples were immediately taken after the transfer (t = 0) and after 6 and 14 h of culture. Two independent experiments were carried out.

To determine hyphal growth on agar plates, a 7-mm plug was inoculated in the center of each 8-cm Petri dish containing MM with the following combination of sugars: glucose, fructose, fructose and glucose, galactose, galactose and fructose, glycerol, glycerol and glucose, lactose, lactose and fructose, xylose, xylose and glucose. Plates were incubated at 22°C for 14 days and colony diameter was measured daily. The data shown are the average of three independent experiments. Error bars indicate standard deviation.

Bacterial host strains were grown in LB medium at 37°C.

### Isolation of the chromosomal *pgi *gene

The chromosomal *pgi1 *gene was isolated from a genomic cosmid library of *T. reesei *(strain VTT-D-80133) in the cosmid p3030 (Hohn & Hinnen, unpublished). The library was screened using a *pgi1 *cDNA fragment as a probe. All positive clones corresponded to the same cosmid. DNA from cosmid K4.1 was digested with enzymes *Stu*I, *Pst*I or *SacI*, blotted and hybridized with a fragment of the *pgi1 *cDNA. Positive fragments of 2.5 and 5 kb from the *Stu*I digestion and the positive 5-kb fragment from the *Pst*I digestion, were subcloned in pBluescriptSK+ (Stratagene, La Jolla, USA). The resulting plasmids were used to clone the gene. ABI PRISM Bigdye™ terminator sequencing kit (Australia) was used to check plasmid constructions and for sequencing the chromosomal *pgi1 *gene.

### Deletion of gene *pgi1 *from the *Trichoderma *genome

The primers used for PCR amplification of *pgi1 *promoter (2.1 kb fragment) from the cosmid clone were Oligo-KpnI-PacI-Prom (+) 5'-CCG AGA GGT ACC**TTA ATT AA**G GCC CCA TCC GTT CTT CCA TGA C-3', where *Kpn*I restriction site is underlined and *Pac*I site is in bold, and Oligo-PGI-KpnI (-) 5'-CGG GGC ATT GGT ACC GTT GGA GAG GTT GTG GTT GAA GTA-3', where *Kpn*I restriction site is underlined. The PCR reaction mixture contained 0.1 U μl^-1 ^Advantage polymerase (BD Biosciences Clontech), 0.2 mM dNTP, 1.1 mM Mg(CH_3_COO)_2_, 10 μM of each primer and 20 ng of the cosmid DNA in a 50 μl final volume. The PCR program used was: 32 cycles of 94°C 25 s, 50°C 3 min, 72°C 3 min, followed by a single cycle of 67°C 7 min. The amplified fragment was isolated from a 10 g l^-1 ^agarose gel, purified through Qiaquick Spin Columns (Qiagen, Netherlands), ligated to TOPO TA cloning vector (Invitrogen) and transformed to TOP10 *E. coli *competent cells. The fragment was isolated from the TOPO vector by digesting with *Asp*718 (isoschizomer of *Kpn*I) (Roche Molecular Biochemicals) and cloned in vector pARO21 (5.7 kb) [64], previously digested with *Asp*718, to obtain plasmid pCL23. The *pgi1 *terminator was amplified by PCR from the cosmid clone as a 1.6-kb fragment using primers Term1.6Forward, 5'-CTGGGGCGTCGAAGCTTGGGCAAGGTCCTT-3' (Restriction site *Hind*III underlined), and Term1.6Reverse, 5'-GGCAAGC**TT****AATTAA**GGC AGGGCGAGCTGAACAA-3' (restriction site *Hind*III is underlined and restriction site *Pac*I is in bold). The conditions for the PCR reaction were as described above. This 1.6-kb fragment contained 0.3 kb of the 3' end of the coding region of the *pgi1 *gene and 1.3 kb of the *pgi1 *terminator. The amplified fragment was digested with *Hind*III and ligated to pCL23, which contained the *pgi1 *promoter and the hygromycin resistance marker to generate plasmid pCL28 (9.5 kb). A fragment of 6.7 kb containing the cassette for deletion was released from pCL28 digesting with *Pac*I and used for transformation of *T. reesei*.

### Transformation procedures

*E. coli *TOP10F' was transformed by electroporation according to the manufacturer's instructions (Bio-Rad). *T. reesei *protoplasts were transformed with 5.5 μg of the *pgi1 *deletion cassette as described earlier [65]. The transformed protoplasts were plated on MM medium where glucose was replaced by 20 g l^-1 ^fructose and 1 g l^-1 ^glucose, and hygromycin (150 μg ml^-1^) was added for selection of the transformants. Monosporic cultures from transformant strains were obtained on selection media (MM medium with 20 g l^-1 ^fructose, 1 g l^-1 ^glucose, 150 μg ml^-1 ^hygromycin and 1 ml l^-1 ^Triton X-100).

Introduction of gene *cre1 *in a Δ*pgi1 *background was done by cotransformation with a 6-kb *Sal*I-*Hind*III genomic fragment from plasmid pMI-41 [17], containing *cre1*, together with plasmid pTOC202. Strains were selected for growth on acetamide in MM with 20 g l^-1 ^fructose, 5 g l^-1 ^glucose and 10 mM acetamide.

The putative transformants were submitted to three selection rounds on selection media to purify them.

### Nucleic acid hybridizations

Genomic DNA of *T. reesei *strains was isolated using Easy DNA™ kit (Invitrogen, CA). Southern blot hybridizations were carried out using standard methods [66]. Probes of gene *pgi1 *for Southern blot analyses were a 1.4 kb fragment of *pgi1 *cDNA obtained from plasmid pMS57 after digestion with *Sac*I, and a 0.6 kb fragment obtained from plasmid pCL5 after digestion with *Pst*I, the latter containing a fragment of the *pgi1 *promoter and the coding region. For detection of the hygromycin resistance gene, either a *Nsi*I-*Xba*I fragment from the plasmid pRLMex30 [20] or a 782-bp *Xba*I-*Sac*II fragment of the hygromycin gene, obtained from plasmid pARO21 [64], were used as probes. The restriction enzymes used for digesting genomic DNA for Southern blot analyses were *BstX*I, *EcoR*I and *Nde*I.

Genomic DNA of wild type QM6a, P40, and 17 putative *cre1^+^*Δ*pgi1 *transformants was digested with *Pst*I to discard transformants without integration of *cre1*. To test the number of integrations of the *cre1 *cassette, genomic DNA from strains QM6a, P40, CP5, CP26 and CP28 was digested with *Eco*RV, *Sac*I and *Xba*I. A 1.2-kb probe corresponding to the complete open reading frame (ORF) was obtained from plasmid pMI-41 with primers 1592: 5'-GGCGGATCCATGCAACGAGCACAGTCTGCC-3' and 1590: 5'-GGCGGATCCCTACATGGCATCCATGAGGTC-3'. A specific probe for a protein-coding region, which did not hybridize with the allele *cre1-1 *of Rut-C30, corresponded to nucleotides +342 to +1209 of *cre1*. This specific probe was amplified from plasmid pMI-41 and DIG-labelled with the PCR DIG probe synthesis kit using primers 1842: 5'-CGCGGATCCAGGCGGGGCAACAAGGGCAG-3', and 1844: 5'-GTGGGAATTCCTACATCCGATCCATGAGGTCGCC-3'. The Southern detection was carried out with detection starter kit II using the standard procedure (Roche Diagnostics GmbH, Mannheim, Germany).

### RNA isolation and expression

Mycelia were lysed in a Fast-Prep^®^-24 homogenizer (MP™Biomedicals LLC Europe, France) using zirconia microbeads by 2 pulses of 30 s at 6 m/s. Total RNA was isolated using the RNeasy Plant Mini kit (Qiagen, Spain). 250 ng of RNA samples were treated with 1 U of rDNAsa I (USB, Affymetrix, Inc) for 15 min at 25°C and the reactions were stopped with 1 μl of 50 mM EDTA and incubated at 65°C for 10 min. Total RNA concentration was estimated using a Nanodrop ND-1000 spectrophotometer (NanoDrop Technologies, Wilmintong, DE, USA).

RT-PCR mixtures contained 1 × Power SYBR-Green PCR Master Mix (Applied Biosystems), 0.125 μl MultiScribe Reverse Transcriptase (50 U μl^-1^), 0.125 μl RNAse Inhibitor (10 U μl^-1^), 100 ng of total RNA and 8 μM of each primer, in a total volume of 25 μl. RT-PCRs were performed in an ABI 7500 (Applied Biosystems). The PCR program consisted of 30 min at 48°C for retrotranscription, 10 min at 95°C, 40 cycles of 15 s at 95°C and 1 min at 60°C. Dissociation curves were done to test amplification validity. Primer sets to analyse gene expression by RT-PCR were: *gpdhF*: 5'-GGGCGGCTACTTTGATGAGTT-3' and *gpdhR*: 5'-TGGTTCTGCATGACGTCTCG-3' for glucose-6-P dehydrogenase gene; *xyrF*: 5'-GAGCTTTCGAGTTCACGCATG-3' and *xyrR*: 5'-CCCAGCAGTACCCGTTGAATT-3' for the xylanase regulator 1, *xyr1 *(AF479644) [21]; *xyl1F*: 5'-CTGTGACTATGGCAACGAAAAGGAG-3' and *xyl1R*: 5'-CACAGCTTGGACACGATGAAGAG-3' [14] for *xy1l*, the gene for D-xylose reductase; *actF*: 5'-TGACATGGCTGGTCGTGATC-3' and *actR*: 5'-ATGTCACGGACGATTTCCCTC-3' for actin-encoding gene (X75421), used as an endogenous control.

Primer design and relative gene expression were done as described by Rodríguez-Ortiz *et al*. [67]. Each RT-PCR analysis was achieved in duplicate to confirm repetitiveness. The values given are the mean of 2 independent experiments with 2 measurements each one. Error bars indicate standard deviation.

### Preparation of cell extracts

Mycelial samples were harvested by filtration through Whatman GF/B glass fiber filters, washed with 7 g l^-1 ^NaCl, frozen in liquid nitrogen and stored at -80°C. Cell extracts from frozen mycelium were prepared as described by Pakula *et al*. [68]. Briefly, equal amounts of mycelia were resuspended in 100 mM Tris buffer pH 7.4, containing 20% glycerol, and 1 tablet of Complete protease inhibitor cocktail (Roche Applied Science, Indianapolis, USA) per 50 ml of buffer. Mycelia were disrupted by 20 cycles of sonication for 8 s followed by 30 s on ice between the cycles and centrifuged at 14,000 × *g *for 10 min. The homogenate was used for enzyme assays.

Protein concentrations of the cell extracts were measured using Bradford method (Bio-Rad, Hercules, CA, USA) using bovine serum globulin (500-0007, Bio-Rad, Hercules, CA, USA) as a standard protein.

### Enzyme assays

Phosphoglucose isomerase (EC 5.3.1.9) was assayed from cell extracts as described by Maitra and Lobo [69]. All enzyme assays were carried out in a Cobas Miras automated analyser (Roche) at 30°C. Phosphoglucose isomerase (PGI) activity was measured by adding the cell extract to a reaction mixture containing 10 mM sodium phosphate buffer (pH 7.0), 0.2 mM NADP^+^, 1 μg glucose-6-P dehydrogenase (Boehringer-Mannheim, GmbH, Germany). The reaction was started adding fructose-6-P (Boehringer-Mannheim, GmbH, Germany) to a final concentration of 2 mM. The activity was calculated from the rate of the decrease in NADH absorbance at 340 nm.

For cellulase activity, culture aliquots were filtered as described above and filtrates were kept at -20°C until measurement. Total cellulase activity in culture supernatants were measured as described by van Tilbeurg [70]. 4-methylumbelliferyl-β-lactoside (MUL) (Sigma, St. Louis, MO, USA) was used as the substrate (0.17 mM). MUL and culture filtrates were incubated in 50 mM sodium acetate buffer (pH 5.0) with a final volume of 100 μl for 5 min at 37°C. The reaction was stopped with 100 μl of 1 M Na_2_CO_3_. Individual activities of cellobiohydrolase (CBH) and endoglucanase were estimated by adding 10 mM cellobiose (inhibits mainly CBH) or 100 mM glucose (inhibits β-glucosidases) to the assay, respectively. Fluorescence was measured on a Varioskan microtiter plate reader (Thermo Scientific, USA) at excitation and emission wavelengths of 350 and 450 nm. Measurements were done from three cultures replicates.

Extracellular β-galactosidase activity was determined in cultures grown in MM with lactose and fructose for 7 days at 30°C and 150 rpm. Supernatants were filtered and concentrated by ammonium sulphate precipitation [71]. The resulting concentrated samples were dialyzed against 3 liters of 50 mM sodium citrate buffer pH 4.5 at 4°C. 200 μl samples were incubated with 850 μl of 50 mM sodium citrate buffer pH 4.8 at 50°C. The reaction was carried out by addition of 150 μl of 10 mM *o*-nitrophenyl-β-D-galactopyranoside as a substrate and incubated for 20-40 min. The reaction was stopped with 500 μl of 1 M Na_2_CO_3_. Activity was measured at 405 nm against a supernatant blank for each sample in a spectrophotometer (Beckman DU^®^, USA). One unit of β-galactosidase activity was defined as the amount of enzyme releasing 1 μmol of *p*-nitrophenyl per min under the conditions used.

Extracellular protein concentration was determined by Bradford method (Bio-Rad, Hercules, CA, USA) using bovine serum globulin (500-0007, Bio-Rad, Hercules, CA, USA) as a standard protein.

### Measurement of sugar concentrations

Fructose and glucose concentrations in culture medium were measured by HPLC (Waters, Milford, Massachusetts, USA) using an Aminex HPX-87H column (Bio Rad Labs, USA) with a 2487 dual α absorbance detector and 5 mM H_2_SO_4 _(Titrisol, Merck) as an eluent at a flow rate of 0.6 ml min^-1 ^at 55°C. Glucose and fructose were used as standards. The data was processed using Water Millenium software.

Lactose and galactose concentrations were analysed enzymaticaly using the Boehringer 176 303 Lactose kit (Boehringer Mannheim, Germany) on the Cobas Miras automated analyser (Roche Applied).

### Microscopy

Samples were observed under a microscope (model BHS-RFK, Olympus Optical Ltd., Tokyo, Japan). The microscope was connected to a video camara (Sensicam, PCO CD Imaging, Kelheim, Germany) and digital image analysis software (analySIS^® ^3.0, Soft Imaging System GmgH, Münster, Germany).

### Statistical analysis

Statistical significance of differences in gene expression among *Trichoderma *strains were evaluated with a Student's *t*-test.

## Abbreviations

Fructose-6-P: fructose 6-phosphate; glucose-6-P: glucose 6-phosphate; GPDH: glucose 6-phosphate dehydrogenase; MUL: 4-methylumbelliferyl-β-lactoside; PGI: phosphoglucose isomerase; PMI: phosphomannose isomerase; PPP: pentose phosphate pathway; ribose-5-P: ribose 5-phosphate.

## Competing interests

The authors declare that they have no competing interests.

## Authors' contributions

MCL designed and performed all the experiments, carried out the data analysis and drafted the manuscript. TP participated in the design of the study, data analysis and drafting of the manuscript. MS participated in the coordination of the study. MP designed the study and helped to draft the manuscript. All authors read and approved the manuscript.

## Supplementary Material

Additional file 1 **Extracellular protein in media with lactose**. Extracellular protein concentration was measured in the supernatants of cultures of Rut-C30, P40 and P-58 in: **(A) **MM with lactose and glucose. **(B) **MM with lactose and fructose. Cultures were grown at 30°C and 200 rpm. Extracellular protein was measured from triplicates of each strain. Errors indicate standard deviation. Click here for file
