# Supplementary figures and images for: The effects of disruption of phosphoglucose isomerase gene on carbon utilisation and cellulase production in Trichoderma reesei Rut-C30
Source: Microb Cell Fact. 2011 May 24;10:40. doi: 10.1186/1475-2859-10-40 (PMC3126698; doi:10.1186/1475-2859-10-40)

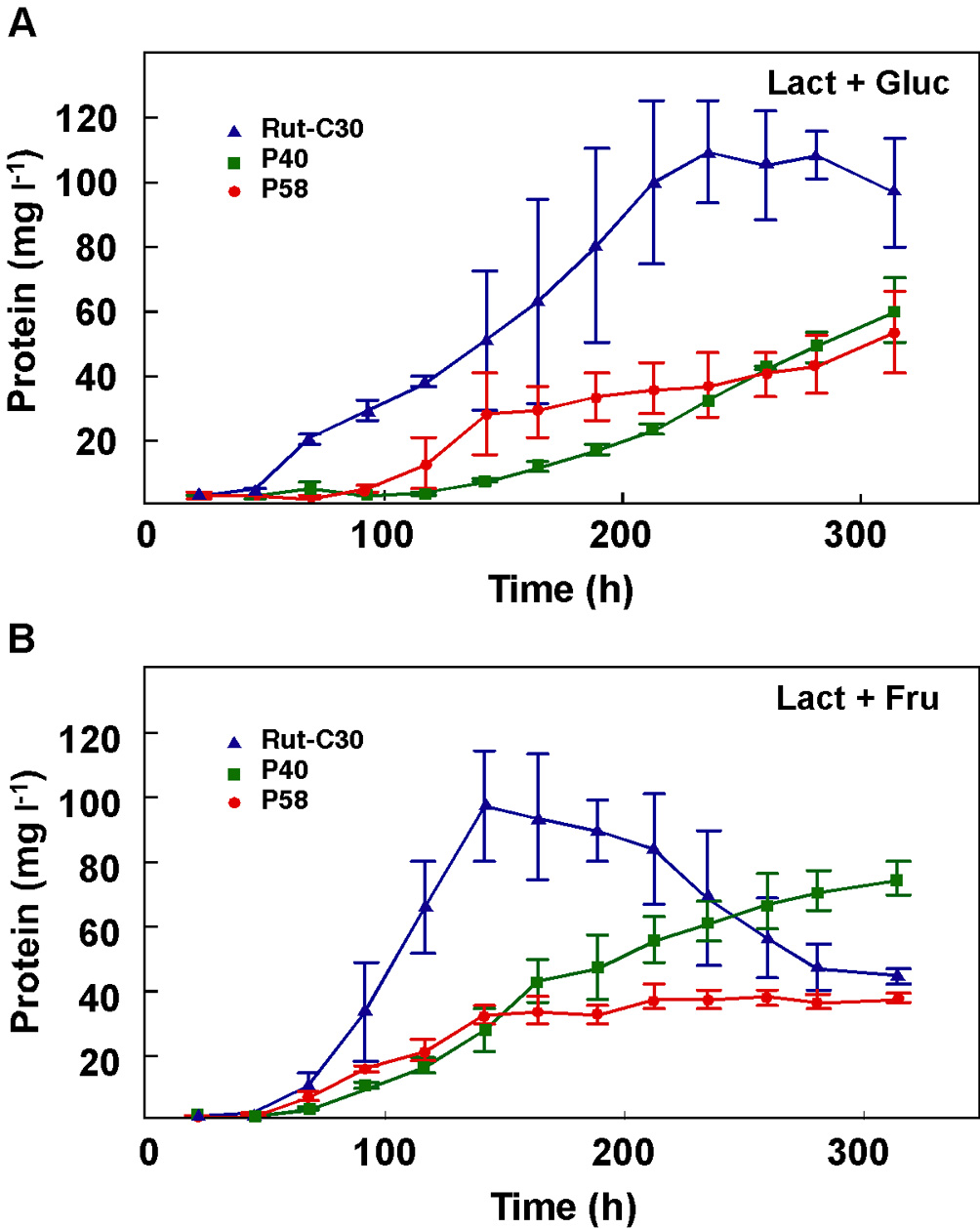

Supplement: Additional file 1 — Extracellular protein in media with lactose. Extracellular protein concentration was measured in the supernatants of cultures of Rut-C30, P40 and P-58 in: (A) MM with lactose and glucose. (B) MM with lactose and fructose. Cultures were grown at 30°C and 200 rpm. Extracellular protein was measured from triplicates of each strain. Errors indicate standard deviation. [file 1475-2859-10-40-S1.JPEG]
